# Peanut gene expression profiling in developing seeds at different reproduction stages during *Aspergillus parasiticus *infection

**DOI:** 10.1186/1471-213X-8-12

**Published:** 2008-02-04

**Authors:** Baozhu Guo, Xiaoping Chen, Phat Dang, Brian T Scully, Xuanqiang Liang, C Corley Holbrook, Jiujiang Yu, Albert K Culbreath

**Affiliations:** 1 USDA-ARS, Crop Protection and Management Research Unit, Tifton, Georgia 31793, USA; 2 University of Georgia, Department of Plant Pathology Tifton, Georgia 31793, USA; 3 USDA-ARS, National Peanut Research Laboratory, Dawson, Georgia 39842, USA; 4 University of Florida, Indian River Research and Education Center, Ft. Pierce, Florida 34945, USA; 5 Guangdong Academy of Agricultural Sciences, Institute of Crop Sciences, Guangzhou, China; 6 USDA-ARS, Crop Genetics and Breeding Research Unit, Tifton, Georgia 31793, USA; 7 USDA-ARS, Southern Regional Research Center, New Orleans, Louisiana 70124, USA

## Abstract

**Background:**

Peanut (*Arachis hypogaea *L.) is an important crop economically and nutritionally, and is one of the most susceptible host crops to colonization of *Aspergillus parasiticus *and subsequent aflatoxin contamination. Knowledge from molecular genetic studies could help to devise strategies in alleviating this problem; however, few peanut DNA sequences are available in the public database. In order to understand the molecular basis of host resistance to aflatoxin contamination, a large-scale project was conducted to generate expressed sequence tags (ESTs) from developing seeds to identify resistance-related genes involved in defense response against *Aspergillus *infection and subsequent aflatoxin contamination.

**Results:**

We constructed six different cDNA libraries derived from developing peanut seeds at three reproduction stages (R5, R6 and R7) from a resistant and a susceptible cultivated peanut genotypes, 'Tifrunner' (susceptible to *Aspergillus *infection with higher aflatoxin contamination and resistant to TSWV) and 'GT-C20' (resistant to *Aspergillus *with reduced aflatoxin contamination and susceptible to TSWV). The developing peanut seed tissues were challenged by *A. parasiticus *and drought stress in the field. A total of 24,192 randomly selected cDNA clones from six libraries were sequenced. After removing vector sequences and quality trimming, 21,777 high-quality EST sequences were generated. Sequence clustering and assembling resulted in 8,689 unique EST sequences with 1,741 tentative consensus EST sequences (TCs) and 6,948 singleton ESTs. Functional classification was performed according to MIPS functional catalogue criteria. The unique EST sequences were divided into twenty-two categories. A similarity search against the non-redundant protein database available from NCBI indicated that 84.78% of total ESTs showed significant similarity to known proteins, of which 165 genes had been previously reported in peanuts. There were differences in overall expression patterns in different libraries and genotypes. A number of sequences were expressed throughout all of the libraries, representing constitutive expressed sequences. In order to identify resistance-related genes with significantly differential expression, a statistical analysis to estimate the relative abundance (*R*) was used to compare the relative abundance of each gene transcripts in each cDNA library. Thirty six and forty seven unique EST sequences with threshold of *R *> 4 from libraries of 'GT-C20' and 'Tifrunner', respectively, were selected for examination of temporal gene expression patterns according to EST frequencies. Nine and eight resistance-related genes with significant up-regulation were obtained in 'GT-C20' and 'Tifrunner' libraries, respectively. Among them, three genes were common in both genotypes. Furthermore, a comparison of our EST sequences with other plant sequences in the TIGR Gene Indices libraries showed that the percentage of peanut EST matched to *Arabidopsis thaliana*, maize (*Zea mays*), *Medicago truncatula*, rapeseed (*Brassica napus*), rice (*Oryza sativa*), soybean (*Glycine max*) and wheat (*Triticum aestivum*) ESTs ranged from 33.84% to 79.46% with the sequence identity ≥ 80%. These results revealed that peanut ESTs are more closely related to legume species than to cereal crops, and more homologous to dicot than to monocot plant species.

**Conclusion:**

The developed ESTs can be used to discover novel sequences or genes, to identify resistance-related genes and to detect the differences among alleles or markers between these resistant and susceptible peanut genotypes. Additionally, this large collection of cultivated peanut EST sequences will make it possible to construct microarrays for gene expression studies and for further characterization of host resistance mechanisms. It will be a valuable genomic resource for the peanut community. The 21,777 ESTs have been deposited to the NCBI GenBank database with accession numbers ES702769 to ES724546.

## Background

Peanut (*Arachis hypogaea *L.) is an important economical crop for oil production and nutritious food for human consumption. However, aflatoxin contamination caused by *Aspergillus *fungi is a great concern in peanut production worldwide. Aflatoxins are the most toxic and carcinogenic compounds associated with both acute and chronic toxicity in animals and humans [1,2]. Both drought stress and high geocarposphere temperature during the latter part of the growing season compromise peanut defense to fungal invasion and exacerbate aflatoxin formation in the seeds [3-6]. Drought stress, extreme temperature or fungal infection can also impair plant growth and yield performance. The development of adapted peanut germplasm and cultivars with improved host-plant resistance is one of our main research objectives.

Resistance to several pathogens is known in peanut [7] indicating that peanuts have evolved a series of defense mechanisms against invasion by plant pathogens. A better understanding of the molecular mechanism for resistance to *Aspergillus *collonization will aid in designing strategies to develop new resistant peanut cultivars. The availability of genomic tools and bio-informatics softwares will significantly improve our ability to a better understanding of the genetic mechanisms of host-plant resistance and to facilitate the genetic improvement of cultivated peanut. Genomic research can also be used to discover novel genes with potential resistance and to develop molecular markers for use in marker-assisted selection. Recently, some genes and proteins associated with *A. parasiticus *or/and drought stress were identified and studied utilizing genomic and proteomic tools [8-12]. With the completion of the rice and *Arabidopsis *whole genome sequencing projects, a vast amount of valuable data has been generated to facilitate cross-species genome comparison in the plant Kingdom. The peanut genome size is significantly larger (2,800 Mb/1C) than the currently sequenced plants [13], such as *Arabidopsis *(128 Mb), rice (420 Mb), and *Medicago *(500 Mb) [14,15]. Financial requirement makes it unrealistic to completely sequence the whole peanut genome in the near future. Therefore, peanut Expressed Sequenced Tags (EST) would be the cost-effective strategy to identify important peanut genes involved in defense to fungal invasion and to study gene expression pattern as well as genetic regulation [16,17].

Expressed Sequence Tags (EST) is an effective genomic approach for rapid identification of expressed genes, and has been widely used in genome-wide gene expression studies in various tissues, developmental stages or under different environmental conditions [18-21]. In addition, the availability of cDNA sequences has accelerated further molecular characterization of genes of interest and provided sequence information for microarray construction and genome annotation [11,22-25]. As of March 23, 2007, large number of ESTs of the top five plant species including *Arapidopsis *(1,276,131), rice (1,211,154), maize (1,161,193), wheat (855,272) and barley (437,728) have been deposited to the GenBank database (dbEST release 032307) [26]. These sequences provide opportunities to accelerate the understanding of the genetic mechanisms that control plant growth and responses to the environment. In contrast, there were only 19,790 *Arachis *ESTs deposited in GenBank, among which 13,226 were derived from cultivated peanut *A. hypogaea *and the remaining 6,264 from the wild species of *A. stenosperma*. These ESTs submitted by different peanut researchers were from different tissues and subjected to different abiotic and biotic stresses [11,27,28].

In this report, an effort for large-scale sequencing of cDNA was carried out with two goals: gene expression comparison between these two genotypes, 'Tifrunner' and 'GT-C20', and providing genomic resource for discovery and understanding of novel defense-related genes involved in resistance to *Aspergillus *colonization and drought stress. To increase gene diversity in the EST population and the probability of identifying genes associated with drought tolerance and disease resistance, different cDNA libraries were prepared from developing seeds at late reproductive stages of a resistant and a susceptible peanut genotypes challenged by *A. parasiticus *and drought stress. Six libraries were constructed that resulted in a total of 21,777 high-quality EST sequences, from which 8,689 unique sequences were identified. To provide useful information on the expression profiling of resistant genes at various seed developmental stages and to offer valuable genomic resource for peanut functional genomics, an extensive analysis of these ESTs was performed using a variety of computational approaches. A functional catalog of expressed genes is reported here as well as a preliminary view of their expression profiles in developing seeds at different developmental stages. This functional catalog seeks to link genes and pathways, and to provide a list of features that could aid in the understanding of how resistance genes are involved in response to biotic and abiotic challenges and how their expression is regulated.

## Results

### Generation of ESTs from developing seeds challenged by *A. parasiticus *and drought stress

**Figure 1:**
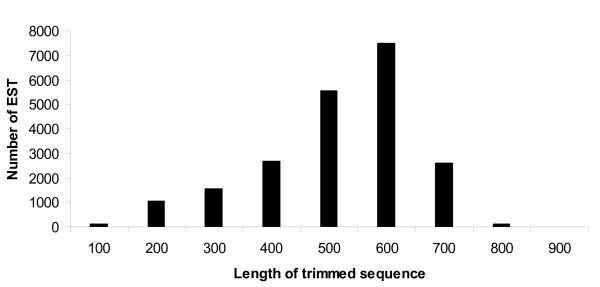
The length of trimmed EST sequence (cDNA length after removal of vector sequence and low quality sequences) submitted to clustering. The number of EST within different categories of trimmed sequence length is presented on the Y-axis. The number on the X-axis represent ranges of trimmed sequence lengths (101–200, 201–300, 301–400 bp, etc, respectively).

Six cDNA libraries were constructed from developing seeds of two varieties ('GT-C20' and 'Tifrunner') collected at three reproductive stages (R5, R6 and R7) after challenging by *A. parasiticus *and drought stress. From the six cDNA libraries, a total of 24,290 clones were randomly selected, sequenced and analyzed using Sequencher software. The vector sequences of the raw sequence reads were trimmed off and low-quality sequences (shorter than 100 bp in length) were removed. A total of 21,777 high-quality EST sequences (about 86%) were generated from the 24,290 clones. Total 8,672 ESTs were generated from 'GT-C20' and 12,426 ESTs were generated from 'Tifrunner' (Table 1). The percentage of acceptable quality EST sequences from individual libraries varied from 81% to 88%. The average length of the ESTs is 411 bp ranging from 114 to 933 bp (Fig. 1). The sum of the total ESTs equal to 8.7 Mb of peanut genome. These quality ESTs combined from both genotypes at three stages were further assembled into 8,689 unique ESTs. Among them, 6,948 were singletons and 1,741 were TCs. The 21,777 ESTs have been deposited to the NCBI GenBank database with accession numbers ES702769 to ES724546.

**Table 1:** Summary of EST sequences, contigs, and singletons in six libraries from 'GT-C20' and 'Tifrunner'

**Library ID**	**Total No. of clones sequenced**	**Accepted sequences (%)**	**No. of TCs (%)**	**No. of Singletons (%)**	**Unique Sequence**
C20R5	5, 184	4, 678 (88)	390 (21)	1, 435 (79)	1, 825
C20R6	2, 304	1, 977 (86)	101 (15)	580 (85)	681
C20R7	2, 496	2, 017 (81)	138 (20)	547 (80)	685
TFR5	7, 104	6, 132 (86)	669 (22)	2, 438 (78)	3, 107
TFR6	4, 800	4, 230 (88)	302 (17)	1, 467 (83)	1, 768
TFR7	2, 304	2, 046 (88)	141 (23)	481 (77)	622

Total	24, 192	21, 098 (86)	1, 741 (20)	6, 948 (80)	8, 688

### Overlapping of unique EST sequences and high redundancy of genes

**Figure 3:**
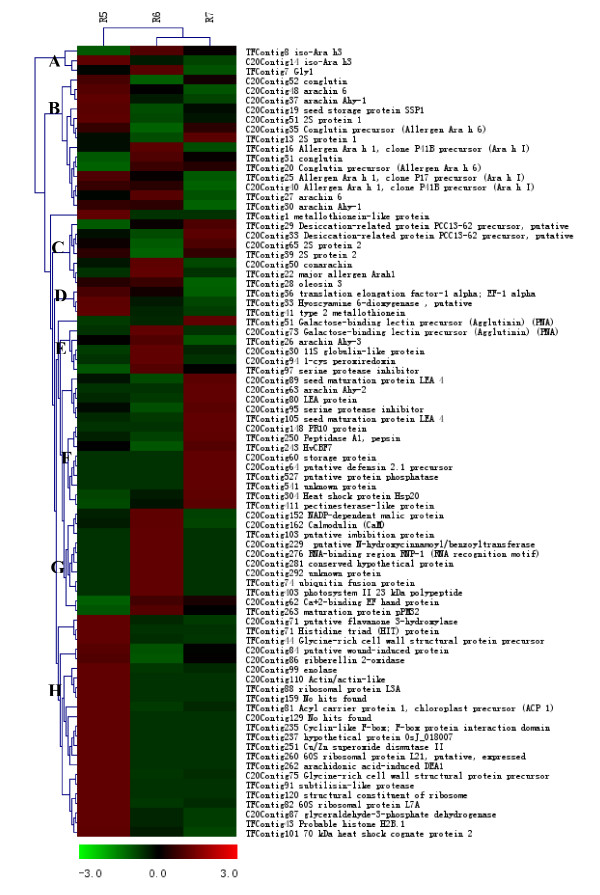
Hierarchical clustering analysis of differentially expressed transcripts for 'GT-C20' and 'Tifrunner'. TCs with R > 4 (84 in total) were used for hierarchical clustering analysis.

A comparison of unique EST sequences from the two genotypes and different stages of developing seeds allows the identification of common and unique sets of expressed genes among the six libraries. The unique ESTs from the six libraries were summarized in Table 1. A total of 1,825, 681, 685, 3,107, 1,768 and 622 unique sequences were present in the C20R5, C20R6, C20R7, TFR5, TFR6 and TFR7, respectively. The distribution and overlapping of these unique EST sequences is shown in Figure 3.

**Figure 2:**
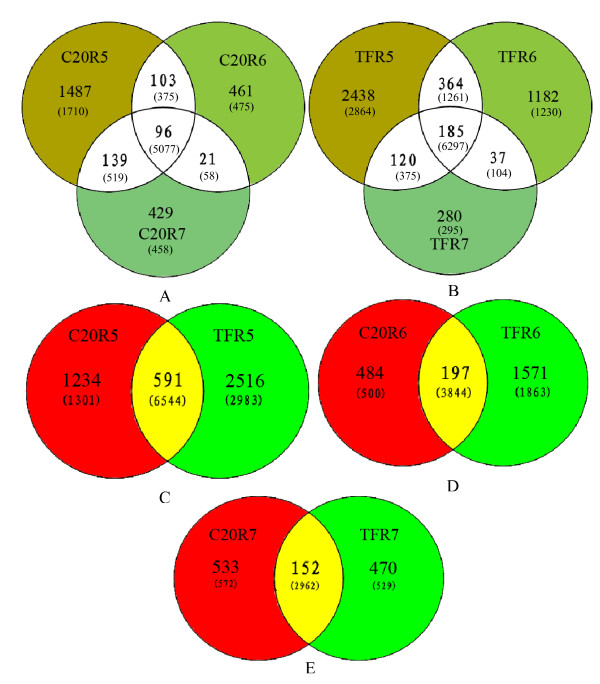
Overlapping of unique peanut EST sequences. A: Common and unique sets of expressed genes among the 'GT-C20' three libraries; B: Common and unique sets of expressed genes among the 'Tifrunner'; C: Common and unique sets of expressed genes between 'GT-C20' and 'Tifrunner' libraries at developmental R5 stage; D: Common and unique sets of expressed genes between 'GT-C20' and 'Tifrunner' libraries at developmental R6 stage; E: Common and unique sets of expressed genes between 'GT-C20' and 'Tifrunner' libraries at developmental R7 stage. The number in the parenthesis presents the number of clones assembled into unique ESTs.

Among the unique ESTs from the C20R5, C20R6 and C20R7 libraries, only 96 ESTs (3%) were shown common to all three libraries (Fig. 2A). The number of ESTs that were common between any two libraries varied from 10.9% to 34.3%. When the same analysis was applied to the ESTs from the TFR5, TFR6 and TFR7, similar results were obtained (Fig. 2B). The ESTs that were common to all three 'Tifrunner' libraries were about 3.4%, similar to that of 'GT-C20'. There were 364 (8%) ESTs that were common to TFR5 and TFR6 libraries, 120 (2.6%) ESTs were found common to both TFR5 and TFR7 libraries, 37 (0.7%) ESTs were found common to both TFR6 and TFR7 libraries. In order to investigate differential gene expression between the resistant and susceptible genotypes, we also performed a comparative analysis between 'GT-C20' and 'Tifrunner' libraries at each seed developmental stage. There were 591 (11.74%), 197 (8.04%) and 152 (11.65%) genes were found common to 'GT-C20' and 'Tifrunner' at R5, R6 and R7, respectively (Fig. 2C, D, and 2E). These results indicated that the differences in transcript abundance might reflect genuine differences in the gene expression in the different libraries. These variations may be due to the differences in disease resistance, tolerance to abiotic stress or other genetic factors at the different developmental stages.

Genes that are shared between or among the libraries included highly expressed transcripts. To further investigate the high frequency of transcripts, all six libraries were analyzed, clustered and assembled individually by genotype. Those highly expressed genes (TCs) assembled from more than 20 individual ESTs were listed in Table 2 for the 'GT-C20' libraries (C20R5, C20R6 and C20R7), and Table 3 for the 'Tifrunner' libraries (TFR5, TFR6 and TFR7). A total of 8,672 ESTs from 'GT-C20' and 12,426 ESTs from 'Tifrunner' non-normalized libraries were assembled into 599 and 1,119 TCs, respectively. There were 27 GT-C20' and 36 'Tifrunner' highly expressed transcripts assembled from more than 20 individual consensus ESTs were selected for distribution analysis (Table 2 and 3). These TCs were concurrently queried against GenBank non-redundant protein database (nr) in searching their putative functions. The BLAST results showed that all the highly expressed genes (TCs) were homologous to known fragments in the GenBank database (Table 2 and 3). There were 31 highly expressed genes, identified by BLAST search, to have the same putative function in both 'GT-C20' and 'Tifrunner' libraries. These highly expressed genes encode constitutive proteins such as allergen protein (C20Contig14 and TFContig8 for iso-Arah3) (Guo et al., unpublished data), storage proteins (C20Contig51 and TFContig31 for 2S protein 1), structural protein (C20Congtig75 and TFContig44 for glycine-rich cell wall structural protein precursor), and stress-resistance associated proteins (C20Contig33 and TFContig29 for desiccation-related protein PCC13-62 precursor).

**Table 2:** Gene expression frequency and BLAST results of the unique ESTs assembled from more than 20 consensus ESTs in the C20R5, C20R6 and C20R7 libraries

				**NCBI BLAST**			
				
**Contig**	**C20R5**	**C20R6**	**C20R7**	**Accession no**.	**Species**	**Gene description**	**E Value**
C20Contig14	369	231	183	gb|ABI17154.1|	*A. hypogaea*	iso-Ara h3	0
C20Contig37	283	123	67	gb|AAU21490.1|	*A. hypogaea*	arachin Ahy-1	0
C20Contig52	205	94	170	gb|AAW56068.1|	*A. hypogaea*	conglutin	6e^-79^
C20Contig47	245	116	95	gb|AAG01363.1|	*A. hypogaea*	Gly1	0
C20Contig35	173	73	165	sp|Q647G9|	*A. hypogaea*	Conglutin precursor (Allergen Ara h 6)	3e^-79^
C20Contig48	192	117	46	gb|ABL14270.1|	*A. hypogaea*	arachin 6	0
C20Contig51	145	74	96	gb|AAU21494.1|	*A. hypogaea*	2S protein 1	9e^-94^
C20Contig40	103	97	44	sp|P43238|	*A. hypogaea*	Allergen Ara h 1, clone P41B precursor (Ara h I)	0
C20Contig19	86	60	70	gb|AAT00598.1|	*A. hypogaea*	seed storage protein SSP1	1e^-104^
C20Contig9	79	41	47	gb|AAU21499.2|	*A. hypogaea*	oleosin 1	1e^-88^
C20Contig34	59	17	25	gb|AAT00596.1|	*A. hypogaea*	conarachin	0
C20Contig57	36	25	26	gb|AAU21501.1|	*A. hypogaea*	oleosin 3	8e^-88^
C20Contig33	21	14	34	gb|ABN09090.1|	*M. truncatula*	Desiccation-related protein PCC13-62 precursor	1e^-106^
C20Contig65	20	15	23	gb|AAU21496.1|	*A. hypogaea*	2S protein 2	5e^-80^
C20Contig50	15	32	8	gb|AAT00597.1|	*A. hypogaea*	conarachin	1e^-169^
C20Contig66	29	6	7	gb|AAZ20291.1|	*A. hypogaea*	metallothionein-like protein	3e^-46^
C20Contig28	24	8	4	gb|AAW56067.1|	*A. hypogaea*	arachin Ahy-4	0
C20Contig74	21	5	3	gb|AAC15413.1|	*O. sativa*	translation elongation factor-1 alpha; EF-1 alpha	0
C20Contig24	16	3	9	gb|AAT00599.1|	*A. hypogaea*	seed storage protein SSP2	3e^-66^
C20Contig71	24	3	0	gb|AAM48133.1|	*S. medusa*	putative flavanone 3-hydroxylase	3e^-65^
C20Contig58	13	9	4	ref|XP_001377994.1|	*M. domestica*	PREDICTED: similar to formin 2	4e^-23^
C20Contig73	6	14	6	sp|P02872|	*A. hypogaea*	Galactose-binding lectin precursor (Agglutinin) (PNA)	1e^-152^
C20Contig68	18	4	2	gb|AAZ20276.1|	*A. hypogaea*	oleosin 1	5e^-70^
C20Contig77	12	6	6	gb|AAU21493.1|	*A. hypogaea*	conarachin	0
C20Contig31	13	6	2	sp|P29828|	*M. sativa*	Protein disulfide-isomerase precursor (PDI)	0
C20Contig4	9	5	6	gb|ABE81150.1|	*M. truncatula*	Major intrinsic protein	1e^-131^
C20Contig75	18	0	3	sp|P27483|	*A. thaliana*	Glycine-rich cell wall structural protein precursor	5e^-06^

**Table 3:** Gene expression frequency and BLAST results of the unique ESTs assembled from more than 20 consensus ESTs in the TFR5, TFR6 and TFR7 libraries

				**NCBI BLAST**			
				
**Contig**	**R5**	**R6**	**R7**	**Accession no**.	**Species**	**Gene description**	**E Value**
TFContig7	250	360	158	gb|AAG01363.1|	A. hypogaea	Gly1	0
TFContig8	104	257	190	gb|ABI17154.1|	A. hypogaea	iso-Ara h3	0
TFContig25	130	119	104	sp|P43237|	A. hypogaea	Allergen Ara h 1, clone P17 precursor (Ara h I)	0
TFContig13	112	90	150	gb|AAU21494.1|	A. hypogaea	2S protein 1	7e^-98^
TFContig31	95	137	119	gb|AAW56068.1|	A. hypogaea	conglutin	3e^-79^
TFContig16	124	230	78	sp|P43238|	A. hypogaea	Allergen Ara h 1, clone P41B precursor (Ara h I)	0
TFContig30	138	135	65	gb|AAU21490.1|	A. hypogaea	arachin Ahy-1	0
TFContig20	89	118	114	sp|Q647G9|	A. hypogaea	Conglutin precursor (Allergen Ara h 6)	6e^-79^
TFContig27	88	126	57	gb|ABL14270.1|	A. hypogaea	arachin 6	0
TFContig35	87	79	34	gb|AAU21499.2|	A. hypogaea	oleosin 1	4e^-90^
TFContig5	54	56	23	gb|AAW56067.1|	A. hypogaea	arachin Ahy-4	0
TFContig28	34	35	27	gb|AAU21501.1|	A. hypogaea	oleosin 3	7e^-88^
TFContig1	56	14	14	gb|AAZ20291.1|	A. hypogaea	metallothionein-like protein	3e^-46^
TFContig29	10	28	40	gb|ABN09090.1|	M. truncatula	Desiccation-related protein PCC13-62 precursor	1e^-106^
TFContig39	32	13	33	gb|AAU21496.1|	A. hypogaea	2S protein 2	3e^-81^
TFContig33	41	13	3	gb|AAT40509.2|	S. demissum	Hyoscyamine 6-dioxygenase, putative	2e^-07^
TFContig41	35	10	5	gb|AAZ20290.1|	A. hypogaea	type 2 metallothionein [Arachis hypogaea]	3e^-45^
TFContig42	27	18	5	gb|ABC75834.1|	G. max	glyceraldehyde-3-phosphate dehydrogenase	0
TFContig36	26	18	1	gb|AAC15413.1|	O. sativa	translation elongation factor-1 alpha; EF-1 alpha	0
TFContig46	20	16	3	gb|AAA99868.1|	G. hirsutum	peroxidase	1e^-170^
TFContig51	8	9	16	sp|P02872|	A. hypogaea	Galactose-binding lectin precursor (Agglutinin) (PNA)	1e-^152^
TFContig4	15	14	1	gb|AAZ20276.1|	A. hypogaea	oleosin 1	7e^-70^
TFContig50	15	12	3	gb|AAC17529.1|	S. saman	aquaporin 2	1e^-154^
TFContig60	15	13	2	gb|ABE80997.1|	M. truncatula	Phosphoglycerate kinase	0
TFContig63	22	6	2	gb|ABM45856.1|	A. hypogaea	cytosolic ascorbate peroxidase	1e^-142^
TFContig48	14	13	1	sp|P29828|	M. sativa	Protein disulfide-isomerase precursor (PDI)	0
TFContig64	14	7	6	gb|AAB84262.1|	A. hypogaea	omega-6 desaturase	0
TFContig65	10	9	8	gb|ABE81150.1|	M. truncatula	Major intrinsic protein	1e^-131^
TFContig66	7	14	6	gb|AAL73404.1|	C. avellana	11S globulin-like protein	1e^-118^
TFContig67	14	7	3	gb|ABF51006.1|	A. hypogaea	Cu-Zn superoxide dismutase	3e^-83^
TFContig44	20	2	1	sp|P27483|	A. thaliana	Glycine-rich cell wall structural protein precursor dbj|BAA94983.1| unnamed protein product	5e^-06^
TFContig61	10	8	5	dbj|BAD99508.1|	V. angularis	gibberellin 2-oxidase	1e^-127^
TFContig70	13	8	1	gb|ABE82912.1|	M. truncatula	Ribosomal protein S4, bacterial and organelle form	1e^-104^
TFContig38	8	8	5	gb|AAM48133.1|	S. medusa	putative flavanone 3-hydroxylase	3e^-64^
TFContig71	19	1	1	gb|ABE83728.1|	M. truncatula	Histidine triad (HIT) protein	3e^-28^
TFContig72	13	7	1	gb|AAS18240.1|	G. max	enolase	0

### Functional classification of unique EST sequences

**Figure 4:**
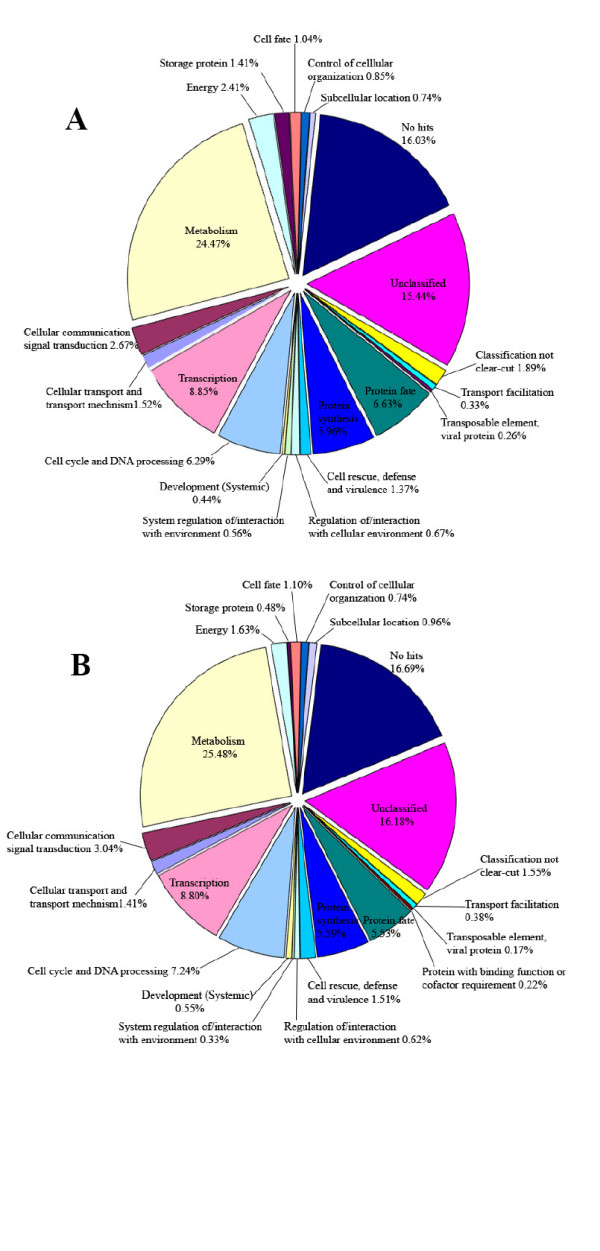
Functional classification of peanut unique ESTs by comparison to Arabidopsis Sequencing Project functional categories. A: functional categories of 'GT-C20' unique EST sequences; B: functional categories of 'Tifrunner' unique ESTs.

In order to further characterize the putative functions of unique ESTs and involvement in different biological processes, a similarity search against the MIPS *Arabidopsis thaliana *Database was performed. According to the MIPS Functional Catalogue criteria, 'GT-C20' unique sequences whose functions could be predicted from the similarity to *Arabidopsis *proteins with an E value of ≤ 1e^-5 ^were classified into twenty-two categories (Fig. 4A) [29,30]. The same analytic procedure was applied to 'Tifrunner' unique ESTs (Fig. 4B). The 'Tifrunner' ESTs with significant protein homology were also sorted into 22 groups. These results suggested that the genes represented by these unique EST sequences may play roles in different biological process.

The results of functional classification showed that the unknown genes, including those which had no hits or low identity (less than 95%) with the *Arabidopsis *protein database and those which matched the unclassified and unknown proteins, represented the largest set of genes (33.33% and 34.42% for 'GT-C20' and 'Tifrunner', respectively). The second largest proportion of genes was found to participate in the biological process of metabolism. The resistance-related and environment-interacted genes were 2.6% and 2.46% in 'GT-C20' and 'Tifrunner', respectively (Fig 4A and 4B). These results indicated that it may be possible to discover novel genes involved in biotic and abiotic responses using the EST profiling startegy.

### Expression profiles of cDNA from different genotypes at different developmental stages

Without normalization or subtraction in library construction, the number of the cDNA clones (or sequenced ESTs) for a given gene reflected the abundance of the gene expression at the corresponding developmental stage. The number of the consensus ESTs that assembled into a unique gene at the three developmental stages may represent the temporal expression pattern of this gene. Therefore, the temporal expression profile of a gene can be deduced by the comparison of the EST frequency at different developmental stage, while the temporal expression profile of a gene of different genotypes may be measured by comparison of the EST frequency of the different genotypes. Given the fact that the absolute EST counts varies in different libraries (Table 1), a meaningful measure of expression profile similarity is independent of these absolute numbers. To test the independence of EST distribution within the libraries, an estimation of the relative abundance defined as *R *(Stekel et al. 2000) was employed to identify the most highly significant differences in EST abundance for each TC among the libraries. The unequal distribution of specific ESTs with statistically significance within each library implied that these ESTs expressed at a higher level in some libraries than others. In order to limit the analysis to those genes which differentially expressed at different developmental stages, only TCs with *R *value larger than 4 were used for hierarchical clustering analysis. This *R *value provided an 82.2% true positive rate [31]. According to the cutoff threshold of *R *> 4, 37 TCs from 'GT-C20' libraries and 47 from 'Tifrunner' libraries were selected to search against GenBank non-redundant protein database (nr) (Table 4 and 5).

**Table 4:** Top hits of C20 unique EST sequences with *R *> 4

				**NCBI BLAST**				
					
**Contig**	**R5**	**R6**	**R7**	***R***	**Accession no**.	**Species**	**Gene description**	**E Value**
C20Contig35	156	69	150	26.01	sp|Q647G9|	*A. hypogaea*	Conglutin precursor (Allergen Ara h 6)	3e^-79^
C20Contig52	205	94	170	20.2	gb|AAW56068.1|	*A. hypogaea*	conglutin	6e^-79^
C20Contig40	103	97	44	17.48	sp|P43238|	*A. hypogaea*	Allergen Ara h 1, clone P41B precursor (Ara h I)	0
C20Contig48	192	117	46	16.71	gb|ABL14270.1|	*A. hypogaea*	arachin 6	0
C20Contig50	15	32	8	16	gb|AAT00597.1|	*A. hypogaea*	conarachin	1e^-169^
C20Contig63	0	0	9	13.13	gb|AAU21491.1|	*A. hypogaea*	arachin Ahy-2	1e^-23^
C20Contig37	283	123	67	12.27	gb|AAU21490.1|	*A. hypogaea*	arachin Ahy-1	0
C20Contig33	21	14	34	11.87	gb|ABN09090.1|	*M. truncatula*	Desiccation-related protein PCC13-62 precursor, putative	1e^-106^
C20Contig14	369	231	183	10.58	gb|ABI17154.1|	*A. hypogaea*	iso-Ara h3	0
C20Contig80	1	0	9	10.49	gb|AAY54009.1|	*A. hypogaea*	LEA protein	2e^-44^
C20Contig71	24	3	0	9.83	gb|AAM48133.1|	*S. medusa*	putative flavanone 3-hydroxylase	3e^-65^
C20Contig19	86	60	70	8.96	gb|AAT00598.1|	*A. hypogaea*	seed storage protein SSP1	1e^-104^
C20Contig148	0	0	6	8.75	gb|AAU81922.1|	*A. hypogaea*	PR10 protein	8e^-67^
C20Contig95	4	0	10	8.68	gb|AAY59891.1|	*A. hypogaea*	serine protease inhibitor	4e^-59^
C20Contig75	16	0	1	7.53	sp|P27483|	*A. thaliana*	Glycine-rich cell wall structural protein precursor	5e^-06^
C20Contig73	6	14	6	6.89	sp|P02872|	*A. hypogaea*	Galactose-binding lectin precursor (Agglutinin) (PNA)	1e^-152^
C20Contig30	3	10	4	6.17	gb|AAL73404.1|	*C. avellana*	11S globulin-like protein	1e^-117^
C20Contig110	10	0	0	6.17	gb|ABE83769.1|	*M. truncatula*	Actin/actin-like	0
C20Contig87	14	2	0	5.57	gb|ABC75834.1|	*G. max*	glyceraldehyde-3-phosphate dehydrogenase	0
C20Contig62	0	4	3	5.51	gb|AAB71227.1|	*G. max*	Ca+2-binding EF hand protein	1e^-113^
C20Contig152	1	5	0	5.31	gb|AAF73006.1	*R. communis*	NADP-dependent malic protein	0
C20Contig65	20	15	23	5.21	gb|AAU21496.1|	*A. hypogaea*	2S protein 2	1e^-79^
C20Contig51	145	74	96	5.17	gb|AAU21494.1|	*A. hypogaea*	2S protein 1	9e^-94^
C20Contig84	13	0	6	4.93	emb|CAB65284.1|	*M. sativa*	putative wound-induced protein	4e^-12^
C20Contig99	11	0	1	4.81	gb|AAS18240.1|	*G. max*	enolase	0
C20Contig86	11	0	6	4.5	dbj|BAD99508.1|	*Vigna angularis*	gibberellin 2-oxidase	1e^-127^
C20Contig229	0	3	0	4.44	ref|NP_851111.1|	*A. thaliana*	putative N-hydroxycinnamoyl/benzoyltransferase	2e^-76^
C20Contig276	0	3	0	4.44	gb|ABE82094.1|	*M. truncatula*	RNA-binding region RNP-1 (RNA recognition motif)	2e^-17^
C20Contig281	0	3	0	4.44	gb|ABE81198.1|	*M. truncatula*	conserved hypothetical protein	3e^-58^
C20Contig292	0	3	0	4.44	ref|NP_567466.1|	*A. thaliana*	unknown protein	1e^-86^
C20Contig60	0	0	3	4.38	gb|AAR02860.1|	*A. hypogaea*	storage protein	5e^-31^
C20Contig64	0	0	3	4.38	gb|AAV85438.1|	*M. sativa*	putative defensin 2.1 precursor	2e^-26^
C20Contig94	3	8	3	4.34	gb|AAT67997.1|	*M. truncatula*	1-cys peroxiredoxin	1e^-105^
C20Contig129	7	0	0	4.32	No hits found			
C20Contig89	3	1	7	4.08	gb|AAG37451.1|	*G. tomentella*	seed maturation protein LEA 4	3e-59
C20Contig162	1	4	0	4.03	sp|P17928|	*M. sativa*	Calmodulin (CaM)	4e-79

**Table 5:** Top hits of TF unique EST sequence with *R *> 4

					**NCBI BLAST**			
					
**Contig**	**R5**	**R6**	**R7**	***R***	**Accession no**.	**Species**	**Genes description**	**E Value**
TFContig8	104	257	190	124.92	gb|ABI17154.1|	*A. hypogaea*	iso-Ara h3	0
TFContig13	112	90	150	69.23	gb|AAU21494.1|	*A. hypogaea*	2S protein 1	7e^-98^
TFContig31	95	137	119	49.24	gb|AAW56068.1|	*A. hypogaea*	conglutin	3e^-79^
TFContig7	250	360	158	48.2	gb|AAG01363.1|	*A. hypogaea*	Gly1	0
TFContig20	89	118	114	46.85	sp|Q647G9|	*A. hypogaea*	Conglutin precursor (Allergen Ara h 6)	6e^-79^
TFContig29	10	28	40	34.14	gb|ABN09090.1|	*M. truncatula*	Desiccation-related protein PCC13-62 precursor, putative	1e^-106^
TFContig16	104	182	58	31.57	sp|P43238|	*A. hypogaea*	Allergen Ara h 1, clone P41B precursor (Ara h I)	0
TFContig25	130	119	104	22.09	sp|P43237|	*A. hypogaea*	Allergen Ara h 1, clone P17 precursor (Ara h I)	0
TFContig27	88	126	57	17.08	gb|ABL14270.1|	*A. hypogaea*	arachin 6	0
TFContig39	32	13	33	16.13	gb|AAU21496.1|	*A. hypogaea*	2S protein 2	1e^-80^
TFContig22	20	48	20	13.98	gb|AAL27476.1|	*A. hypogaea*	major allergen Arah1	1e^-172^
TFContig105	2	1	11	13.30	gb|AAG37451.1|	*G. tomentella*	seed maturation protein LEA 4	2e^-56^
TFContig91	16	0	0	11.09	gb|AAQ23176.1|	*G. max*	subtilisin-like protease	1e^-168^
TFContig51	8	9	16	9.81	sp|P02872|	*A. hypogaea*	Galactose-binding lectin precursor (Agglutinin) (PNA)	1e^-152^
TFContig120	13	0	0	9.01	ref|NP_187143.1|	*A. thaliana*	structural constituent of ribosome	2e^-63^
TFContig71	19	1	1	8.08	gb|ABE83728.1|	*M. truncatula*	Histidine triad (HIT) protein	3e ^-28^
TFContig82	13	0	1	7.23	ref|NP_001061550.1|	*O. sativa*	60S ribosomal protein L7A	1e^-132^
TFContig250	1	0	5	7.09	gb|ABD32384.1|	*M. truncatula*	Peptidase A1, pepsin	1e^-131^
TFContig44	20	2	1	7.04	sp|P27483|	*A. thaliana*	Glycine-rich cell wall structural protein precursor	5e^-06^
TFContig1	56	14	14	6.6	gb|AAZ20291.1|	*A. hypogaea*	metallothionein-like protein	3e^-46^
TFContig33	41	13	3	6.43	gb|AAT40509.2|	*S.demissum*	Hyoscyamine 6-dioxygenase, putative	2e^-07^
TFContig88	9	0	0	6.24	gb|AAQ96335.1|	*N. tabacum*	ribosomal protein L3A	1e^-125^
TFContig159	9	0	0	6.24	No hits found			
TFContig304	0	1	4	5.86	gb|ABD32352.1|	*M. truncatula*	Heat shock protein Hsp20	4e^-63^
TFContig43	14	2	0	5.85	sp|Q1S9I9|	*M. truncatula*	Probable histone H2B.1	2e^-71^
TFContig28	34	35	27	5.81	gb|AAU21501.1|	*A. hypogaea*	oleosin 3	7e^-88^
TFContig30	138	135	65	5.72	gb|AAU21490.1|	*A. hypogaea*	arachin Ahy-1	0
TFContig527	0	0	3	5.46	ref|NP_001062774.1|	*O. sativa*	putative protein phosphatase	1e^-105^
TFContig541	0	0	3	5.46	gb|AAL87284.1|	*A. thaliana*	unknown protein	4e^-15^
TFContig103	0	5	0	5.43	emb|CAB71135.1|	*C. arietinum*	putative imbibition protein	1e^-125^
TFContig26	6	11	0	5.06	gb|AAU21492.1|	*A. hypogaea*	arachin Ahy-3	0
TFContig243	2	0	4	4.84	gb|AAX23704.1|	*H. vulgare*	HvCBF7	3e^-44^
TFContig36	26	18	1	4.81	gb|AAC15413.1|	*O. sativa*	translation elongation factor-1 alpha; EF-1 alpha	0
TFContig97	2	8	5	4.61	gb|AAY59891.1|	*A. hypogaea*	serine protease inhibitor	4e^-59^
TFContig74	0	4	0	4.34	gb|AAZ20285.1|	*A. hypogaea*	ubiquitin fusion protein	1e^-67^
TFContig403	0	4	0	4.34	emb|CAA41713.1|	*N. tabacum*	photosystem II 23 kDa polypeptide	1e^-72^
TFContig411	0	1	3	4.29	emb|CAB82677.1|	*A. thaliana*	pectinesterase-like protein	5e^-47^
TFContig81	8	0	1	4.22	sp|P93092|	*C. glauca*	Acyl carrier protein 1, chloroplast precursor (ACP 1)	4e^-40^
TFContig235	6	0	0	4.16	gb|ABE77917.1|	*M. truncatula*	Cyclin-like F-box; F-box protein interaction domain	1e^-47^
TFContig237	6	0	0	4.16	gb|EAZ34524.1|	*O. sativa*	hypothetical protein OsJ_018007	2e^-23^
TFContig251	6	0	0	4.16	emb|CAA39819.1|	*P. sativum*	Cu/Zn superoxide dismutase II	7e^-89^
TFContig260	6	0	0	4.16	gb|ABF93903.1|	*O. sativa*	60S ribosomal protein L21, putative, expressed	3e^-83^
TFContig262	6	0	0	4.16	emb|CAI51313.1|	*C. chinense*	arachidonic acid-induced DEA1	3e^-25^
TFContig263	0	4	2	4.16	gb|AAD49719.1|	*G. max*	maturation protein pPM32	2e^-32^
TFContig41	35	10	5	4.12	gb|AAZ20290.1|	*A. hypogaea*	type 2 metallothionein	3e^-45^
TFContig101	12	3	0	4.07	gb|AAS57913.1|	*V. radiata*	70 kDa heat shock cognate protein 2	0

Based on the abundance and the *R *statistic, a clustering analysis was performed to assess the relatedness of each library in terms of gene expression profiles. As Ewing et al. (1999) described [32], we compiled the 84 TCs into a matrix file comprised of the frequency of ESTs corresponding to each contig in the library that represented different seed developmental stages and performed hierarchical clustering analysis. From hierarchical clustering analysis, the 84 TCs with different redundant and similar expression patterns could be grouped into eight major clusters from A to H as shown in Figure 4. Each cluster represents a different expression profile. Hierarchical clustering analysis showed that most of high abundant genes with same putative functions from 'GT-C20' libraries and 'Tifrunner' libraries could be grouped into the same cluster. These genes usually encode constitutive proteins (such as arachin, conglutin and oleosin) and their expression patterns are not genotype dependent. Some putative genes related to resistance such as PR10 protein and defensin 2.1 precursors were found only in 'GT-C20' and the expression pattern was up-regulated (Fig. 3).

The results of hierarchical clustering and similarity search indicated that the 84 unique ESTs (*R *> 4) with similar DNA sequence were not equally distributed between the 'GT-C20' and 'Tifrunner' libraries. In comparison, only 32 unique ESTs (*R *> 4) were not equally distributed within different 'GT-C20' libraries (Table 4 and Fig. 3). There were seven, ten and eight unique TCs were observed in the C20R5, C20R6 and C20R7 libraries, respectively. Three unique TCs (C20Contig40 for allergen Ara1, C20Contig48 for arachin 6 and C20Contig37 for arachin Ahy-1) were observed between C20R5 and C20R6 libraries. These three unique EST contigs (C20Contig35 for conglutin precursor, C20Contig52 for conglutin and C20Congtig86 for gibberellin 2-oxidase) were primarily found in the C20R5 and C20R7 libraries. Only one unique EST (C20Contig62 for Ca^+2^-binding EF hand protein) had cDNA clones represented only in C20R6 and C20R7 libraries. Four unique ESTs (C20Contig14 for iso-Ara h3, C20Contig19 for seed storage protein SSP1, C20Contig65 for 2S protein 2 and C20Contig51 for 2S protein 1) had cDNA clones equally distributed across the three libraries of 'GT-C20'.

In the three 'Tifrunner' libraries, there were 38 unique ESTs (*R *> 4) whose cDNA clones were not equally distributed (Table 5 and Fig. 3). Comparison within all 'Tifrunner' libraries, fourteen, five and seven unique EST sequences were observed in TFR5, TFR6 and TFR7 libraries, respectively. Six unique ESTs were observed only in TFR5 and TFR6 but absent in TFR7 libraries. Two unique ESTs were predominately present in the TFR6 and TFR7. The remaining unique ESTs with *R *> 4 had cDNA clones equally distributed across the three 'Tifrunner' libraries.

*Defense-related genes identified by database search *

The information provided by ESTs from plant tissues challenged by specific biotic and abiotic stress conditions offered an opportunity for gene discovery. The unique EST sequences from 'GT-C20' and 'Tifrunner' were compared individually to the non-redundant protein sequence database available from NCBI by BLASTx program with a minimum *E *cutoff value < 1e^-5^. In reference to the results of differential expression and hierarchical clustering analysis (Table 4 and 5), only those genes whose expression were significant up or down regulated at different stages were selected. The other defense-related genes whose E value > 1e^-5 ^treated as false positive and were excluded from the analysis.

Among the unique EST sequences with *R *> 4, only three up-regulated putative defense-related genes (putative desiccation-related protein PCC13-62 precursor, serine protease inhibitor and seed maturation protein LEA 4) were identified in both 'GT-C20' and 'Tifrunner' libraries (Table 6 and Fig. 3). Six up-regulated unique EST sequences were observed only in 'GT-C20' libraries, and matched previous reported known protein including PR10 protein, defensin protein and calmodulin (Table 6). In the 'Tifrunner' libraries, five defense-related genes such as metallothionein-like protein, heat shock protein and Cu/Zn superoxide dismutase II were detected with significant up-regulation.

**Table 6:** Putative resistance-related genes with significantly differential expression (*R *> 4) in 'GT-C20' and 'Tifrunner' libraries

Putative Gene function	Organism	'GT-C20'	'Tifrunner'
Desiccation-related protein PCC13-62 precursor, putative	*M. truncatula*	+	+
seed maturation protein LEA 4	*G. tomentella*	+	+
metallothionein-like protein	*A. hypogaea*	-	+
Heat shock protein Hsp20	*M. truncatula*	-	+
serine protease inhibitor	*A. hypogaea*	+	+
Cu/Zn superoxide dismutase II	*P. sativum*	-	+
type 2 metallothionein	*A. hypogaea*	-	+
70 kDa heat shock cognate protein 2	*V. radiata*	-	+
LEA protein	*A. hypogaea*	+	-
PR10 protein	*A. hypogaea*	+	-
Ca+2-binding EF hand protein	*G. max*	+	-
putative wound-induced protein	*M. sativa*	+	-
putative defensin 2.1 precursor	*M. sativa*	+	-
Calmodulin (CaM)	*M. truncatula*	*+*	*-*

+: the putative resistance-related gene was identified in the libraries.

-: no putative resistance-related gene was identified in the libraries.

### Comparison of these EST data to other plant EST sequences

In order to compare these peanut ESTs to other publicly available plant ESTs, a similarity search against several plant EST databases in TIGR Gene Indices was performed (Table 7). When DNA sequence identity was at ≥ 90%, the percentages of peanut ESTs matching soybean and *Medicago truncatula *were 16.45% and 9.82%, respectively. When DNA sequence identity was decreased to ≥ 80%, the percentages of peanut ESTs matched to soybean and *M. truncatula *greatly increased to 79.46% and 72.53%, respectively. In contrast, the percentages of peanut ESTs that matched to *Arabidopsis*, rape seed, rice, maize and wheat ESTs were less than 50%, ranging from 33.84% to 45.69%, when DNA sequence identity was set at ≥ 80%. Although peanut and rape seed are both oilseed crops, when the DNA sequence identity was set at ≥ 80%, the similarity of peanut ESTs matching rape seed ESTs was only 38.5%, far less than that of the legume crops soybean and *M. truncatula*. As expected, peanut ESTs showed a higher similarity to ESTs of the legume species than to those of cereal crops, and also present a higher homology to ESTs of the dicot plants than to those of the monocots.

**Table 7:** Peanut unique EST homologs identified in soybean, *Medicago truncatula*, *Arabidopsis*, rapeseed, rice, maize and wheat in TIGR gene indices

**TIGR Gene Indices**	**Number of ESTs matched to TIGR Gene Indices (Percent in Parentheses)^a^**	
	
	**Identity ≥ 80%**	**Identity ≥ 90%**
Soybean (*Glycine max*)	6904 (79.46)	1429 (16.45)
*Medicago truncatula*	6302 (72.53)	853 (9.82)
*Arabidopsis thaliana*	3970 (45.69)	470 (5.41)
Rapeseed (*Brassica napus*)	3345 (38.50)	465 (5.35)
Rice (*Oryza sativa*)	3128 (36.00)	484 (5.57)
Maize (*Zea mays*)	2716 (31.26)	402 (4.63)
Wheat (*Triticum aestivum*)	2940 (33.84)	469 (5.40)

^a^The criteria for stand-alone BLASTn were: (1) extract-match bp ≥ 11; (2) E value ≤ 1e-5; and (3) identity ≥ 80% and 90% at DNA sequence level.

## Discussion

Larger-scale sequencing of Expressed Sequence Tags (EST) is an effective method for gene discovery. The available peanut EST database in GenBank is 19,790 entries as of March 23, 2007, which were derived from leaf, root, pod, cotyledon and other tissues of cultivated peanut (13,526) and wild species (6,264), respectively. Compared to maize, wheat, rice and soybean, the number and scale of peanut ESTs deposited in GenBank are far behind those major crops and it is inadequate to meet the need of peanut genetic and genomic research. Many successful EST projects have been reported for a number of species and from a variety of tissues under various conditions [6,11,17,27,33,34]. However, most of these EST projects were restricted to different tissues from one genotype or different tissues from different genotypes. The EST project reported in this study is uniquely and systematically designed using the same tissues (developing seeds) from two genotypes, 'GT-C20' and 'Tifrunner' with different characters in terms of resistance and susceptibility to diseases, under the same environmental conditions (challenged by *A. parasiticus *and drought stress) at specific seed developmental stages (R5, R6 and R7). The completion of this peanut EST project makes the available peanut ESTs in the GenBank database doubled for the research community to share. In addition, the six libraries were neither normalized nor subtracted so that the frequency of a unique EST (gene) within each stage could be determined and could provide a hint for the expression level of that specific gene.

To understand the molecular basis of host resistance *to A. flavus/parasiticus *and consequent aflatoxin contamination, we monitored the transcript changes at these three developmental stages in developing seeds. The 8,689 unique ESTs were categorized into different functional groups based on the MIPS criteria [29,30]. The highly expressed overlapping ESTs also helped in assembling full-length unique transcripts expressed in peanut seed, such as the putative allergen protein (iso-Ara h3, GenBank accession no. DQ855115). The putative functions of those identified unique ESTs have been predicted by similarity search according to MIPS (Fig. 4). Comparing to the *Arabidopsis *sequence data, 65.99% of total peanut unique ESTs matched *Arabidopsis *protein sequences with a known function and 17.58% had significant similarity to *Arabidopsis *protein sequences with unknown function. About 16.43% of the total unique ESTs showed no significant similarity to *Arabidopsis *al all. Those peanut ESTs matched *Arabidopsis *know functions were divided into nineteen categories [29,30]. A major portion of these genes with known functions fall in the category of metabolism (24.47%) followed by transcription (8.85%, Fig. 4). To further identify novel peanut sequences, a comprehensive similarity search against GenBank non-redudant (nr) database using the stand-alone BLASTx algorithm was performed and resulted in the identification of an additional 967 putative novel sequences including 165 unique peanut ESTs matching reported known peanut genes. The BLAST result revealed that significant number of unique peanut seed ESTs match soybean (396), *Arabidopsis *(2952), rice (682), and other plant species.

In this study, some previously reported defense-related genes have been confirmed to be expressed. Desiccation-related proteins could be induced by drought stress and were relatively sensitive to cellular dehydration [35,36]. The LEA (late embryogenesis abundant) proteins are known to be involved in protecting higher plants from damage caused by environmental stresses, especially dehydration from drought [37-39]. Serine protease inhibitors are involved in plant defense against pathogens and could be induced in response to infection by pathogens [40-42]. These three different classes of genes were up-regulated in the three reproduction stages of both 'GT-C20' and 'Tifrunner' libraries. Other related-genes with significant differential expression were present either in 'GT-C20' or in 'Tifrunner'. For example, the PR10 protein family is induced by plants in response to pathogen infection as well as abiotic stress, and showed transcriptional up-regulation upon biotic and abiotic stresses [43-45]. Calmodulin (CaM) is a ubiquitous Ca^2+ ^sensor found in all eukaryotes and has been shown to participate in the regulation of diverse calcium-dependent physiological processes [46]. Calmodulin plays an important role in sensing and transducing changes in cellular Ca^2+ ^concentration in response to several biotic and abiotic stresses [47]. CaM has been implicated in plant-pathogen interactions [48,49]. PR10 and Calmodulin were significantly up-regulated in 'GT-C20' libraries but not in 'Tifrunner' (Table 6). In contrast, two heat shock proteins, synthesized in response to heat stress [50-52], were detected up-regulated in 'Tifrunner' libraries but not in 'GT-C20' (Table 6). This raises questions of why certain genes are present or absent or show differential expression in different genotypes, such as 'GT-C20' and 'Tifrunner'. There are two possible hypothetic explanations. One is that in this study we randomly selected clones for cDNA sequencing and might have missed some clones that could be in 'GT-C20' or 'Tifrunner' libraries. The other is that the presence, absence or significantly differential expressions of certain genes, especially defense-related genes, are a result of the genetic differences (resistance and susceptibility) of these two genotypes. In order to verify the assumption that variability of expression might be a result of genetic differences in disease resistance or stresses tolerance, two genes (an allergen protein *iso ara h3*, highly abundant and a constitutively expressed genes, and an LEA 4, a up-regulated and defense-related gene) were selected for sequence similarity analysis. As expected, the similarity of *iso ara h3 *between 'GT-C20' and 'Tifrunner' was 97%, however, *LEA 4 *sequences shared only 91% identity over 709 bases. For *iso ara h3*, among 1,692 consensus sequences, 6 gaps were found. For *LEA 4*, among 709 consensus sequences, 19 gaps were found (data not shown). The results implied that the allelic differences of defense-related genes were higher than that of constitutively expressed genes. Further investigations are necessary to characterize their gene functions and to analyze the patterns of their gene expressions.

## Conclusion

This is a unique study using both resistance and susceptibilities genotypes under the same environmental conditions as challenged by *A. parasiticus *and drought stress at specific seed developmental stages (R5, R6 and R7). The large number of peanut ESTs obtained provides an important resource for gene discovery, for gene expression profiling, and for microarray design [12,53]. The frequency of the individual EST demonstrated the temporal expression patterns of a given gene. The information from this study will significantly improve our understanding the mechanism of host resistance and provide a useful genomic resource for peanut breeding and aflatoxin research community.

## Methods

### Libraries construction and sequencing

The peanut varieties 'Tifrunner', susceptible to *A. parasiticus *but resistant to TSWV (tomato spotted wilt virus, the No.1 disease in southeastern US) and 'GT-C20', resistant to *Aspergillus parasiticus *but susceptible to TSWV, were selected for this experiment. The peanut plant materials used for RNA extraction were grown in the field and inoculated by *A. parasiticus *NRRL 2999 at mid-bloom (60 days after planting). Drought stress was imposed during the final 40 days before harvest through the use of rain-out shelters. Immature pods at the R5 (beginning seed), R6 (full seed) and R7 (beginning maturity) stages [54] from two peanut genotypes, 'GT-C20' and 'Tifrunner', were collected, frozen in liquid nitrogen, and stored at -80°C until RNA extraction.

Developing seeds were removed from the sampled immature pods for total RNA extraction. Six cDNA libraries from developing seeds were constructed according to the protocol reported previously [55]. The cDNA inserts were ligated to the pBlueScript vector. Each of the six cDNA libraries was named using first 2 letters from genotype followed by corresponding developing stage. For example, TFR5 refers to 'Tifrunner' at developing stage R5, and so on.

Sequencing was performed using ABI 3730xl Genetic analyzer (Applied Biosystems) with the ABI Prism BigDye terminator cycle sequencing kit (Foster City, CA) from 5' end of cDNA using T3 sequencing primer.

### EST processing and clustering

The short vector sequences were trimmed off from the raw sequence reads and the poor-quality sequences (less than 100 nucleotides) were removed by the Sequencher 4.6 software (Gene Codes, Ann Arbor, MI). The cleaned cDNA sequences from 'GT-C20' and 'Tifrunner' were separately assembled into TCs through the use of Phrap [56] with 90% minimum match. Sequences sharing greater than 90% identity over 40 or more contiguous bases with unmatched overhang less than 30 bases in length were placed into clusters. Overlaps exclusively on low complexity regions were excluded.

### Frequency of cDNAs in different libraries

The six cDNA libraries were neither normalized nor subtracted. Therefore, the number of cDNA clones comprised of contigs may represent gene expression profiles at the different developmental stage. An "electronic Northern" was conducted through analyzing the frequency of cDNA clones within each contig. Six libraries were divided into two groups for analysis according to source genotype. Either group including three libraries constructed from the same peanut genotype at different stage was separately compiled and analyzed. Each of the three libraries represented different developmental stages (R5, R6 and R7) which were subjected to different lengths of fungal challenge and drought stress was analyzed to identify cDNAs whose presence was specific to that developmental stage and environmental challenge.

### Functional annotation of unique ESTs and bioinformatics

In order to identify the putative functions of unique ESTs by BLAST against the NCBI (National Center for Biotechnology Information) non-redundant protein database (nr) and the Munich Information Center for Protein Sequences (MIPS), *Arabidopsis *Sequencing Project functional categories [29,30] were downloaded and localized.

A sequence similarity comparison between EST sequences and nr database was performed using the BLASTx algorithm [57,58] with NCBI default parameters. The unique sequences were considered to be homologous to known proteins in nr database when the *E *value of BLAST was less than 10^-5 ^(the probability that alignment would be generated randomly is 1<100,000) and the BLAST score was higher than 200. The putative full-length protein-coding region was determined by complete open read frame (ORF), poly (A) and significant similarity to known protein sequence. Functional classifications from MIPS were assigned to each unique EST by referring to MIPS functional catalogue. Resistance/defense-related genes were identified in the ESTs via a combination of similarity to known genes and transcript expression profiles.

Gene expression analysis was performed using TIGR MultiExperiment Viewer software [59] by using transcript abundance in each contig in all six libraries. The significant differences in EST abundance for each contig among the libraries were assessed by an *R *statistic described by Stekel et al. (2000). Only those TCs with *R *> 4 were used for hierarchical clustering analysis.

Comparative genome analysis between our ESTs and the currently available major crop EST gene indice in the databases was performed. These include *Arabidopsis thaliana *(81,826 ESTs), rape seed (*Brassica napus*) (25,929 ESTs), maize (*Zea mays*) (115,744 ESTs), *Medicago truncatula *(36,878 ESTs), rice (*Oryza sativa*) (181,796 ESTs), soybean (*Glycine max*) (63,676 ESTs), and wheat (*Triticum aestivum*) (122,282 ESTs). These TIGR EST gene indice (currently curated at Harvard University) were downloaded from the FTP site [60]. The following criteria were used in BLAST with the TIGR gene index, *E*-value less than 1e-5 and DNA identity more than 80% and 90%.

## Authors' contributions

BZG conceived of the study was responsible for its design, participated in its coordination and cDNA library construction, and drafted and revised the manuscript. XC performed the data analysis, bioinformatics and helped to draft the manuscript. PD performed the library construction, sequencing and data analysis. BTS participated in the sequencing and coordination. XL participated in the design and collected the samples. CCH participated in the design, the field study and sample preparation. JY participated in the sequencing analysis. AKC participated in the field evaluation. All authors have read and approved the final manuscript.
